# Amino acid substitutions at the HIV-1 transframe region significantly impair virus infectivity

**DOI:** 10.1371/journal.pone.0262477

**Published:** 2022-01-27

**Authors:** Fu-Hsien Yu, Kuo-Jung Huang, Chin-Tien Wang

**Affiliations:** 1 Institute of Clinical Medicine, National Yang-Ming University, Taipei, Taiwan; 2 Institute of Clinical Medicine, National Yang Ming Chiao Tung University, Taipei, Taiwan; 3 Division of Clinical Research, Department of Medical Research, Taipei Veterans General Hospital, Taipei, Taiwan; Consejo Superior de Investigaciones Cientificas, SPAIN

## Abstract

A transframe region within HIV-1 Gag-Pol (referred to as p6* or p6pol), directly linked to the protease (PR) N-terminus, plays a pivotal role in modulating PR activation. To identify specific p6* residues involved in PR activation, we created a series of p6* mutants by making substitutions for conserved p6* residues. Our results indicate that some p6* mutants were defective in terms of virus infectivity, despite displaying a wild-type virus particle processing pattern. Mutations at p6* F8 reduced virus infectivity associated with insufficient virus processing, due in part to impaired PR maturation and RT packaging. Our data strongly suggest that conserved Phe (F) residues at position 8 of p6* are involved in the PR maturation process.

## Introduction

**Fig 1:**
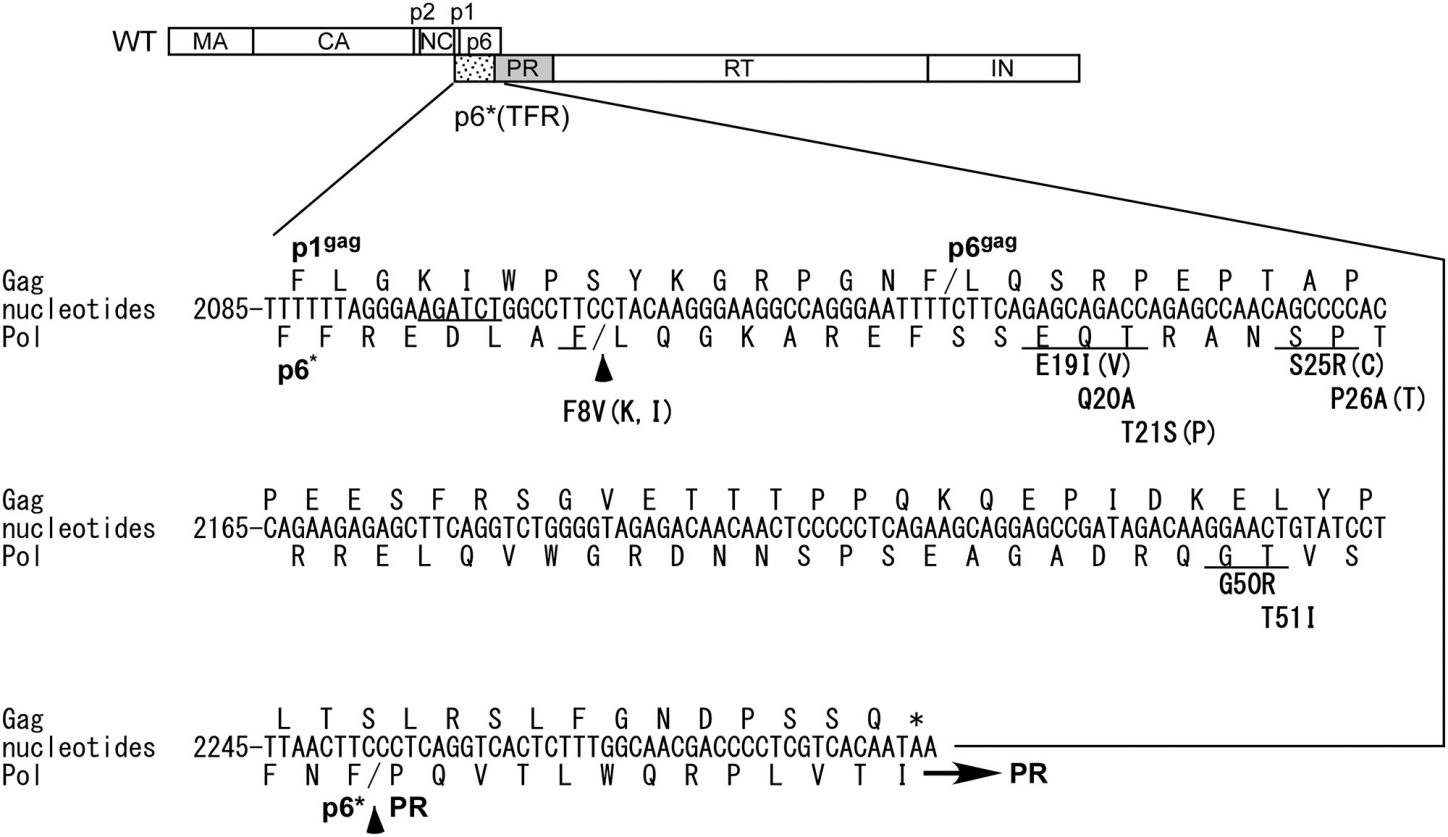
Schematic representation of HIV-1 Gag and Gag-Pol constructs. Indicated are HIV-1 Gag protein domains MA (matrix; p17), CA (capsid; p24), NC (nucleocapsid; p7), p1, p6, pol-encoded transframe region (TFR) p6*, PR, RT and IN. An overlapping reading frame sequence between p1gag-p6gag and p6* and encoded amino acid residues are shown. Positions of mutated amino acid residues are underlined. Alternative substitution residues are in parentheses. Arrowheads indicate cleavages at the p6*/PR junction and the F8/L9 putative internal cleavage site.

Essential retroviral proteases (PRs) are encoded by part of the pol gene [1]. Due to a partial overlap between gag and pol reading frames, HIV-1 Pol is translated as a Gag-Pol fusion protein via a -1 ribosomal frameshift mechanism that occurs at a frequency of 5–10% during Gag translation [2]. Gag-Pol dimerization triggers the activation of embedded PR, and the activated PR becomes a fully functional PR dimer following autocleavage from Gag-Pol [3]. Cleavage of the viral structural Gag precursor Pr55^*gag*^ by PR yields four major products: matrix (MA; p17), capsid (CA; p24), nucleocapsid (NC; p7), and C-terminal p6gag [4]. Two spacer peptides, p2 and p1, separate NC from CA and p6gag, respectively. The proteolytic processing of Gag-Pol produces PR, reverse transcriptase, and integrase (IN) in addition to Gag cleavage products. Within Gag-Pol, p1gag-p6gag is truncated and replaced with a transframe region (TFR) referred to as p6* or p6pol (Fig 1). PR-mediated Gag and Gag-Pol processing (referred to as virus maturation) is essential for viral infectivity [5, 6].

Since premature PR activation triggers premature Gag cleavage prior to Gag multimerization and virus particle formation (resulting in markedly reduced virus yields), temporal and spatial PR activation regulation is critical to virus assembly [7–9]. Several lines of evidence suggest that p6*, located adjacent to the PR N-terminus, plays a modulating role in PR activation. First, p6* removal from PR precursor is required for PR activity to either fully undergo autocleavage or mediate virus particle maturation [10–13]. Second, p6*-derived peptides are capable of blocking PR activity *in vitro* [14, 15]. Molecular model studies further suggest that C-terminal p6* residues may contribute to delayed PR activation by destabilizing PR dimer interface interactions [16–19]. We previously reported that PR dimerization enhancement following p6* replacement with a leucine zipper dimerization motif leads to markedly reduced virus yields due to improved Gag cleavage by PR. However, the presence of a C-terminal p6* tetrapeptide can counteract the Gag cleavage enhancement incurred by a leucine zipper insertion in the deleted p6* region [20–22].

Single amino acid substitutions of the four C-terminal p6* residues can significantly impair virus maturation, strongly suggesting that the C-terminal p6* tetrapeptide is important for PR activation regulation [10, 21]. Data from a study involving stepwise cluster substitutions for p6 codons (3–13 residues) suggest that both C- and N-terminal p6* regions are important, with the middle p6* part being largely dispensable in terms of PR activation [13]. Consistent with these data, a deletion of approximately 63% of p6* amino acid residues, or partial substitution of p6* with a heterologous peptide, does not significantly affect HIV-1 infectivity [23]. Results from our previous study, in which we performed single-cycle-infection assays of HIV-1 virions produced from Gag and Gag-Pol co-transfectants, indicate a significant reduction (20–40% of wt level) in virus infectivity following a major deletion of the p6* coding sequence [21, 24]. The remaining C-terminal p6* tetrapeptide in the p6*-deleted mutant is insufficient for mitigating the reduced infectivity resulting from deficient virus processing [24]. The presence or absence of undefined p6* residues contributing to PR activation regulation is unknown. PR inhibitors in current use largely target mature PR, but this is frequently accompanied by the emergence of PR-resistant HIV-1 variants. Understanding how p6* (especially certain specifically identified p6* residues) contributes to PR activation may support the development of an alternative HIV/AIDS treatment strategy involving p6* or p6*-PR targeting. To identify specific p6* residues involved in PR activation, we created a series of p6* mutants with site-directed mutagenesis of conserved p6* residues without affecting encoded p6gag residues. Mutation effects on virus and PR maturation were analyzed by immunoblot and single-round-infection assays.

## Materials and methods

### Plasmid construction

The parental HIV-1 proviral sequence in this study is HXB2 [25]. All constructs expressing Gag and Gag-Pol were derived from a replication-defective HIV-1 expression vector, HIVgpt [26]. All constructs including the p6* residue substitution mutants (see Table 1) were engineered by PCR-based overlap extension mutagenesis using HIVgpt as a template. We used a downstream reverse primer 5’-GGTACAGTCTCAATAGGGCTAATG-3’ (nt. 2577–51) or an upstream forward primer 5’-AATGATGCAGAGAGGCAAT-3’ (nt.1916-34). The amplified fragments were digested with BglII and BclI, and subcloned into a plasmid cassette pBRCla-Sal that contains HIV-1 coding sequence (from ClaI-nt.831 to SalI-nt.5786). Each mutation-containing pBRCla-Sal cassette was then digested with SpeI and SalI, and ligated into HIVgpt. The resultant constructs were confirmed by DNA sequencing.

**Table 1:** Primers used for creating p6* residue substitution mutations.

No.	Mutation clones	Forward / Reverse primer(5’-3’)
1	F8V	F: 5’-GAA GAT CTG GCC **GTC** CTA CAA GGG AAG-3’
		R: 2578–52 5’-ACT GGT ACA GTC TCA ATA GGG CTA ATG-3’
2	F8K	F: 5’-GAA GAT CTG GCC **AAG** CTA CAA GGG AAG-3’
		R: 2578–52 5’-ACT GGT ACA GTC TCA ATA GGG CTA ATG-3’
3	F8I	F: 5’-GAA GAT CTG GCC **ATC** CTA CAA GG-3’
		R: 2578–52 5’-ACT GGT ACA GTC TCA ATA GGG CTA ATG-3’
4	E19V	F: 5’-G GAA TTT TCT TCA **GTC** CAG ACC AGA GCC AAC AG-3’
		R: 5’-CT GTT GGC TCT GGT CTG **GAC** TGA AGA AAA TTC C-3’
5	Q20S	F: 5’-G GAA TTT TCT TCA GAG **TCG** ACC AGA GCC AAC AG-3’
		R: 5’- CT GTT GGC TCT GGT **CGA** CTC TGA AGA AAA TTC C-3’
6	E19I/Q20A	F: 5’-G GAA TTT TCT TCA **ATC GCG** ACC AGA GCC AAC AG-3’
		R: 5’-CT GTT GGC TCT GGT **CGC GAT** TGA AGA AAA TTC C-3’
7	E19I/Q20A/T21S	F: 5’-G GAA TTT TCT TCA **ATC GCG TCC** AGA GCC AAC AG-3’
		R: 5’-CT GTT GGC TCT **GGA CGC GAT** TGA AGA AAA TTC C-3’
8	E19V/Q20A/T21P	F: 5’-G GAA TTT TCT TCA **GTC GCG CCC** AGA GCC AAC AG-3’
		R:5’-CT GTT GGC TCT **GGG CGC GAC** TGA AGA AAA TTC C-3’
9	S25R	F: 5’-CC AGA GCC AAC **CGC** CCC ACC AGA AGA G-3’
		R: 5’-C TCT TCT GGT GGG **GCG** GTT GGC TCT GG-3’
10	P26A	F: 5’-CC AGA GCC AAC AGC **GCC** ACC AGA AGA G-3’
		R: 5’-C TCT TCT GGT **GGC** GCT GTT GGC TCT GG-3’
11	S25C/P26T	F: 5’-CC AGA GCC AAC **TGC ACC** ACC AGA AGA G-3’
		R: 5’-C TCT TCT GGT **GGT GCA** GTT GGC TCT GG-3’
12	G50R	F: 5’-CC GAT AGA CAA **AGA** ACT GTA TCC TTT A-3’
		R: 5’-T AAA GGA TAC AGT **TCT** TTG TCT ATC GG-3’
13	T51I	F: 5’-CC GAT AGA CAA GGA **ATT** GTA TCC TTT A-3’
		R: 5’-T AAA GGA TAC **AAT** TCC TTG TCT ATC GG-3’
14	G50R/T51I	F: 5’-CC GAT AGA CAA **AGA ATT** GTA TCC TTT A-3’
		R: 5’-T AAA GGA TAC **AAT TCT** TTG TCT ATC GG-3’

^†^ Bold-faced nucleotides denote the mutated codons. BglII sites are underlined. Cloning procedures are described in Materials and Methods.

### Cell culture and transfection

HEK-293T or HeLa cells were maintained in DMEM supplemented with 10% fetal calf serum. Confluent HEK-293T cells were trypsinized, split 1:10 and seeded onto 10-cm plates 24 hours before transfections. For each construct, HEK-293T cells were transfected with 20 μg of plasmid DNA by the calcium phosphate precipitation method [27], with the addition of 50 μm chloroquine to enhance transfection efficiency. Culture media and cells are harvested for protein analysis at 48–72 h post-transfection.

### Immunoblot

Culture media from transfected HEK-293T cells were filtered (0.45-μm pores) and centrifuged through 2 ml 20% sucrose. Viral pellets and cell lysates mixed with sample buffer were subjected to 10% SDS-PAGE as described previously [20]. For separation of HIV-1 PR-associated products, samples were subjected to 4 to 12% bis-Tris gradient gels (NuPage bis-Tris minigels; Thermo Fisher Scientific) and electroblotted onto nitrocellulose membranes as described [28]. HIV-1 Gag proteins were probed with an anti-p24^*gag*^ monoclonal antibody (mouse hybridoma clone 183-H12-5C). For HIV-1 RT detection, the primary antibody is rabbit antiserum or a mouse anti-RT monoclonal antibody [29, 30]. Membrane bound HIV-1 PR was detected with a mouse anti-PR monoclonal antibody (Abcam; ab8327) or a sheep antiserum. Cellular β-actin was detected using a mouse anti-β-actin monoclonal antibody (Sigma). The secondary antibody is a sheep anti-mouse or a donkey anti-rabbit (HRP)-conjugated antibody and the procedures used for HRP activity detection followed the manufacturer’s protocol (Thermo Fisher Scientific).

### Single-cycle infection assays

For infections, 10 μg of wt or each of p6* mutant plasmid plus 5 μg of the VSV- G protein expression plasmid pHCMV-G [31] were co-transfected into HEK-293T cells. Forty-eight hours after transfection, virus-containing supernatant were collected, filtered, and aliquots of the filtrate were diluted to infect HeLa cells in the presence of 4 μg/ml polybrene. The remaining supernatant and cell samples were prepared and subjected to 10% SDS-PAGE. Twenty-four hours after infection, cells were trypsinized, split into dishes and refed with medium containing drug selection cocktail [32]. Selected mycophenolic acid resistant colonies were fixed and stained with 50% methanol containing 0.5% methylene blue. Numbers of drug-resistant colonies are converted into titers (cfu/ml). Infectivity was expressed as the ratio of the mutant titers to wt titers in parallel experiments.

## Results

To identify specific p6* residues involved in PR activation, highly conserved residues believed to be replaceable without affecting encoded p6gag residues were altered using PCR-mediated site-directed mutagenesis. p6* sequences in published HIV-1 strains were aligned, with non-polymorphic residues treated as highly conserved residues (https://www.hiv.lanl.gov/content/sequence/HIV/COMPENDIUM/2006_7/5.pdf). Primers used to generate mutants are shown in Table 1. Of the 14 p6* residue substitution mutants created, 9 contained single amino acid residue substitutions, 3 contained double substitutions, and 2 contained triple substitutions. A four-residue substitution mutant designated E19I/Q20A/T21S/S25G was generated during the PCR-mediated cloning of the E19I/Q20A/T21S mutant. This unintentionally created mutation did not affect encoded p6gag residues.

**Fig 2:**
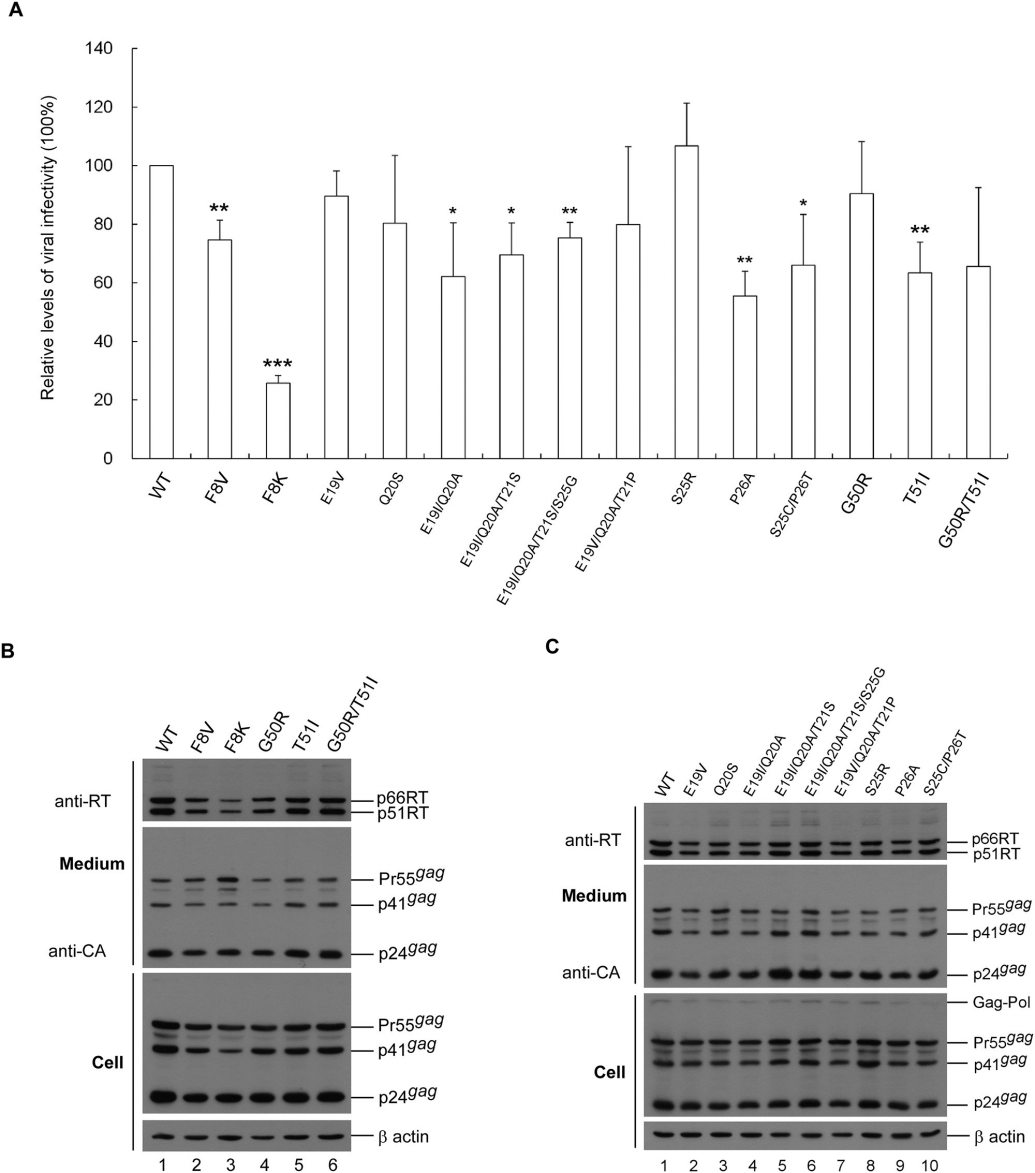
Effects of p6* mutations on virus particle processing and infectivity. HEK293T cells were transfected with a wt HIVgpt or designated p6* mutant construct plus a VSV-G expression vector. Cells and supernatants were collected 48 h post-transfection. Collected and filtered supernatant aliquots were used to infect HeLa cells. Remaining supernatants were used for immunoblot. (A) Relative virus infectivity. Drug-resistant colony infection and selection was performed as described in Materials and Methods section. Mutant infectivity was determined as the ratio of mutant titers to wt titers, normalized to Gag protein levels in parallel experiments. *p < 0.05; **p < 0.05; ***p < 0.001. All results are from three independent experiments. (B-C) Shown is a representative immunoblot. Membrane-bound proteins were initially probed with anti-RT serum prior to stripping and probing with an anti-p24CA monoclonal antibody. HIV-1 Gag-Pol, Pol, 66/51RT, Pr55^*gag*^, p41^*gag*^ and p24^*gag*^ positions are shown.

Impacts of p6* mutations on virus maturation were assessed using single-cycle infection assays and immunoblot of released virions following the transient expression of each mutant with a VSV-G expression vector in HEK-293T cells. According to assay results, some single amino acid substitution mutations exerted small or moderate impacts on virus infectivity, and simultaneous substitutions for two, three or four residues led to noticeable reductions in virus infectivity. For example, E19V or Q20S mutations did not significantly impair virus infectivity, but E19I/Q20A, E19I/Q20A/T21S and E19I/Q20A/T21S/S25G did. Reduced E19V/Q20A/T21P infectivity was statistically non-significant (Fig 2A). Likewise, reduced T51I infectivity became statistically non-significant following the additional replacement of Arg for G50 (G50R/T51I). The individual and multiple substitutions of the p6* residues (such as E19V, E19I/Q20A and E19I/Q20A/T21S, etc.) were selected from databases excluding the polymorphic p6* codons. It remains to be determined if an individual single mutation such as E19I, Q20A, T21P, T21S or S25G has any impact on virus infectivity.

Regarding mutations at the putative internal cleavage site F8/L9, we observed that a Val (V) or Lys (K) replacement at F8 significantly reduced virus infectivity. Specifically, F8K exhibited a five-fold decrease in virus infectivity compared to the wt in parallel experiments. Western blot analysis data indicate that despite defective infectivity, most mutants displayed a virus particle processing profile similar to that of the wt (Fig 2B and 2C). However, some mutants (including F8K) exhibited higher virus-associated Pr55^*gag*^ levels compared to the wt (Fig 2B, lane 3 vs. lane 1), suggesting a virus maturation defect.

### Mutations at a putative internal p6* cleavage site trigger defects in PR maturation and RT incorporation

**Fig 3:**
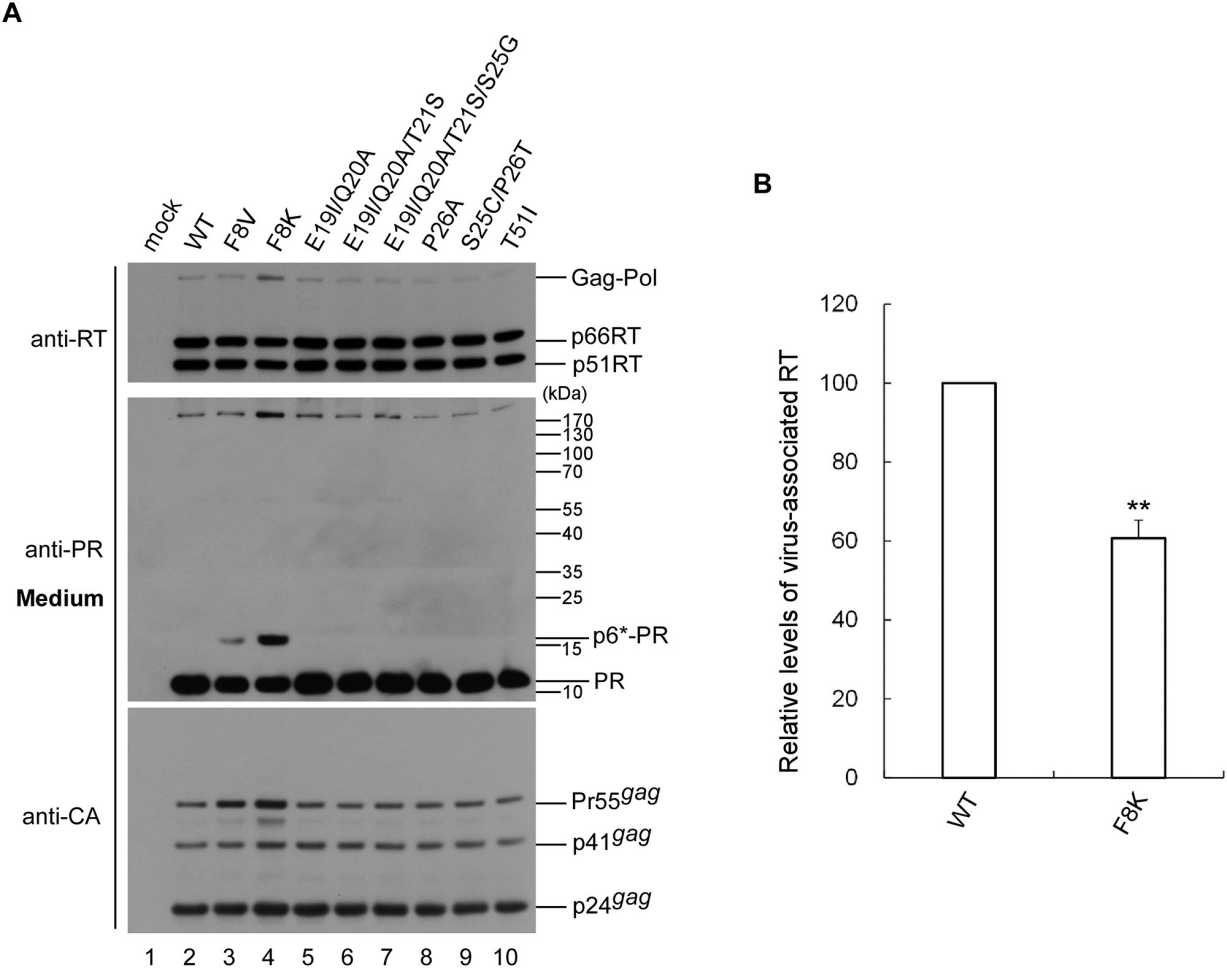
Mutations at the internal p6* cleavage site impaired PR maturation and RT incorporation. HEK293T cells were transfected with designated constructs. Cells and supernatants were collected 48 h post-transfection and subjected to immunoblot. To detect PR-associated products, supernatant samples were separated by 4–12% Bis-Tris gradient gels. Membrane-bound proteins were initially probed with anti-PR serum, stripped, and probed with anti-RT serum, followed by probing with anti-p24CA monoclonal antibodies. Molecular weight size markers (in kDa) are indicated on right side (second upper panel). HIV-1 Gag-Pol, Pol, 66/51RT, p6*-PR, PR, Pr55^*gag*^, p41^*gag*^ and p24^*gag*^ positions are shown. Asterisks indicate 14 kDa PR-associated intermediate precursor positions. (B) Levels of HIV-1 Gag proteins Pr55, p41 and p24, and RT-associated Gag-Pol and p66/51 in each sample were quantified by scanning band densities on immunoblots. Ratios of total Pol versus Gag proteins levels were calculated, and normalized to wt in parallel experiments. Data were obtained from three independent experiments. **p < 0.05.

Given the pivotal role of p6* in modulating the PR activation process, it is likely that the reduced infectivity observed in some p6* mutants may have been due to a defect in the PR activation process, leading to subsequent impairments in virus maturation. To test this possibility, all infectivity-defective mutants were subjected to immunoblot with an HIV-1 PR antiserum. Our data readily detected mature PR in all mutant particles. In addition to mature PR, bands corresponding to the PR precursor (p6*-PR) were observed for F8V and F8K, with substantial amounts of F8K PR present in precursor form (Fig 3A, lanes 3 and 4). Since F8V and F8K exhibited higher levels of virus-associated Pr55gag compared to the wt, impaired PR maturation may have contributed, at least in part, to insufficient virus processing (Fig 3A middle panel, lanes 3 and 4 vs. lane 2). In addition, we found substitution mutations at F8, in particular, F8K markedly reduced virus-associated RT levels (Fig 3B). These data suggest that a native Phe (F) at position 8 of p6* is important for PR maturation and RT incorporation.

Since F8/L9 has been proposed as an internal p6* cleavage site [15, 33, 34], changes in F8 residues may block internal p6* cleavage, thus contributing to impaired PR maturation. If it exists, a cleavage at F8/L9 would supposedly produce a PR intermediate (designated delTFP-p6*-PR) with an 8-amino acid transframe peptide (TFP) removed from the p6*-PR. Since the delTFP-p6*-PR migrates slightly faster than the p6*-PR, the two may be detected by immunoblot as a double band [35]. Accordingly, it is likely that inhibiting p6*-PR autocleavage by reducing PR activity might allow for the detection of p6*-PR and delTFP-p6*-PR in wt virions. To test this possibility, wt and F8K transfectants were mock-treated or treated with low doses of an HIV-1 PR inhibitor. An F8I mutation (referred to as F440I in the Pettit group’s reports) is capable of blocking the F8/L9 internal p6* cleavage site in synthesized Gag-Pol *in vitro* [36, 37]. We used an F8I mutant to serve as a control.

**Fig 4:**
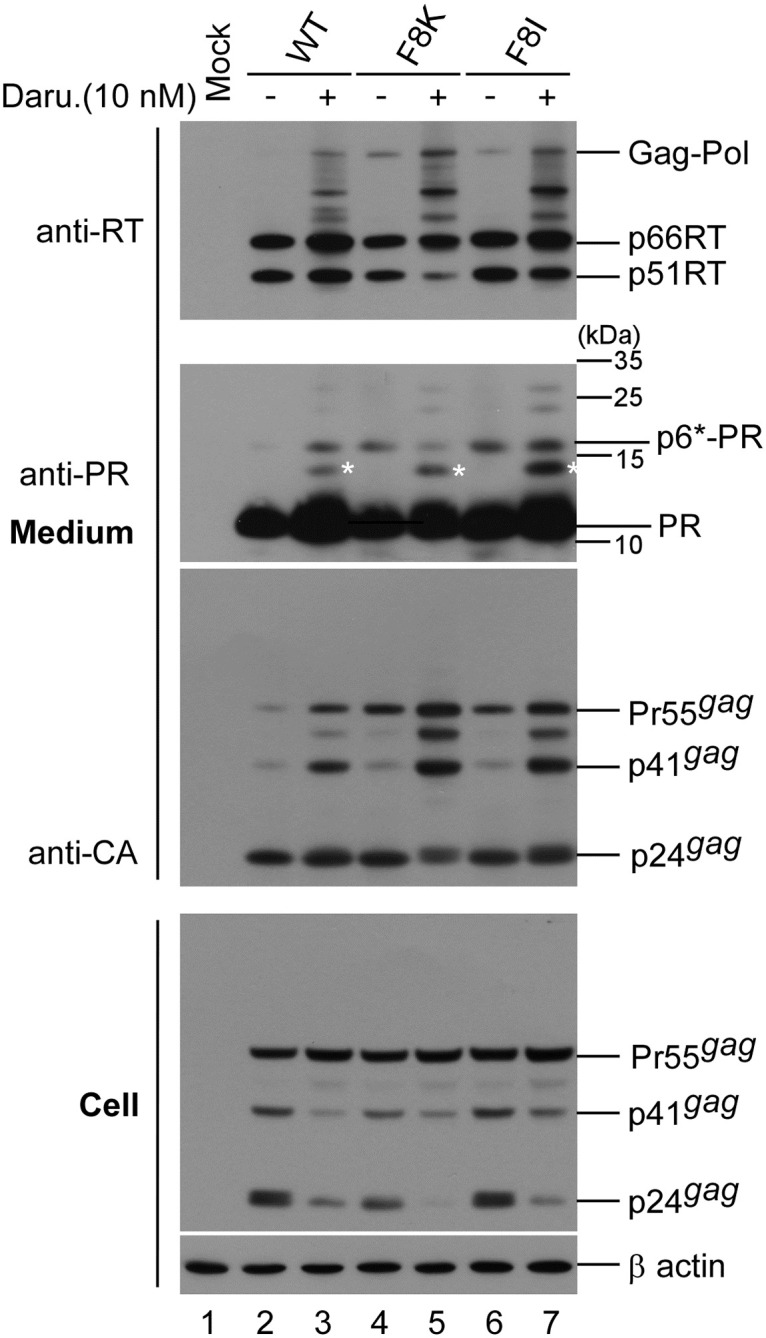
Effects of partial PR activity inhibition on PR maturation. HEK293T cells were transfected with designated construct. At 4 h post-transfection, equal amounts of cells were plated on two dishes and either left untreated or treated with darunavir (an HIV-1 protease inhibitor) at a concentration of 10 nM. Supernatants and cells were collected 48 h post-transfection, prepared, and subjected to immunoblot as described in the Fig 3 caption. Asterisks indicate 14 kDa PR intermediate positions. Shown is a representative immunoblot from three independent experiments.

Our results indicate that both F8I and F8K exhibited higher levels of virus-associated Pr55gag compared to the wt (Fig 4, lanes 4 and 6 vs. lane 2), suggesting a virus particle processing defect. Unexpectedly, we did not observe a double band of PR precursors; instead, two distinct immature PR products (17 kDa and 14 kDa) were readily detected in wt samples when PR activity was partially inhibited (Fig 4, lane 3). This suggests that F8/L9 cleavage may occur very rapidly, or that the degree of partial PR activity inhibition completely blocks F8/L9 cleavage. Unlike the wt, both F8K and F8I had readily detected bands corresponding to p6*-PR, regardless of PR inhibition treatment or no treatment (Fig 4, lanes 4–7). The PR-associated 14 kDa products were likely derived from a cleavage at a cryptic site within p6*-PR.

**Fig 5:**
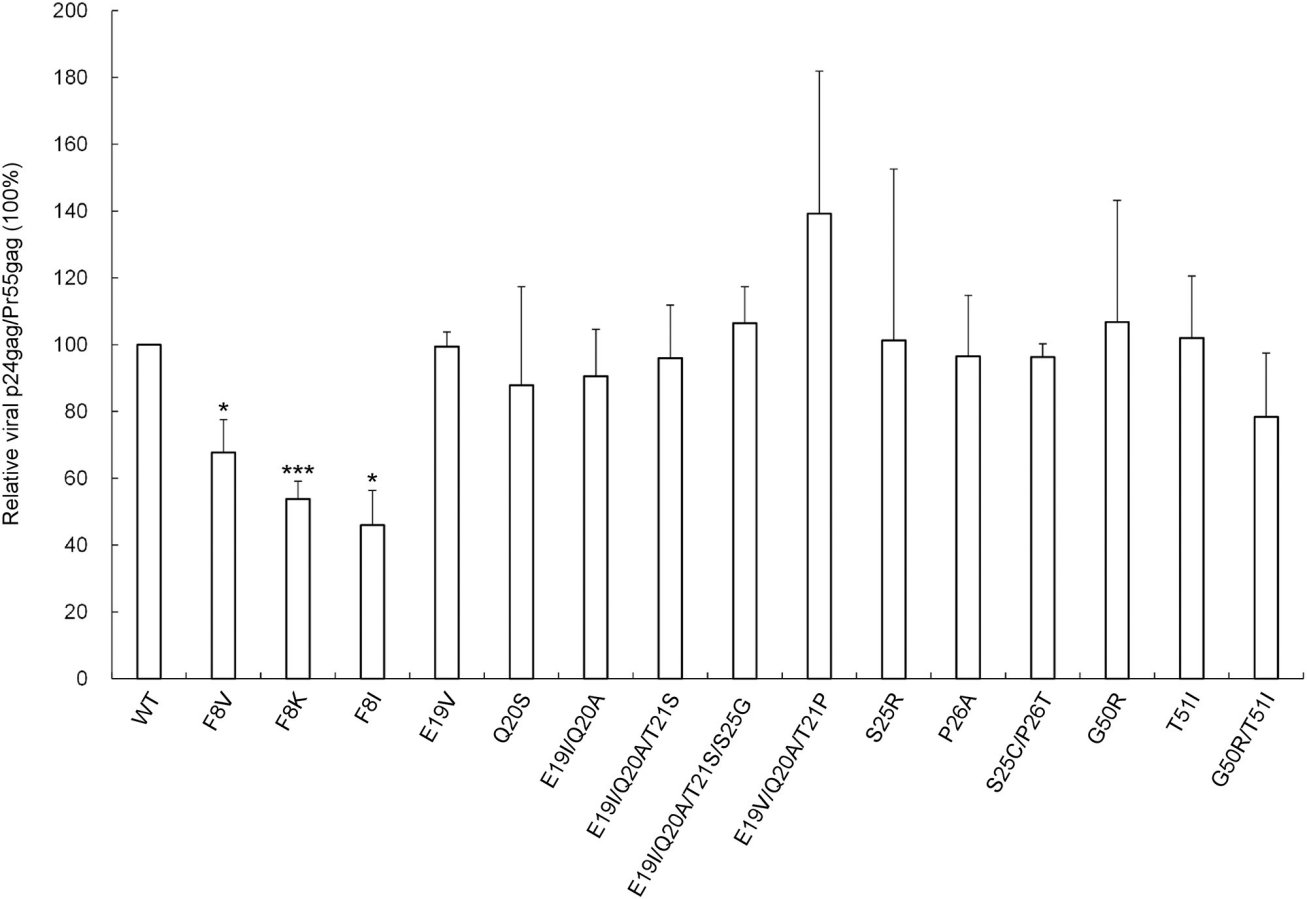
Virus particle processing efficiency data for p6* mutants. Virus-associated Pr55^*gag*^ and p24^*gag*^ levels were quantified as immunoblot band densities. Ratios of p24^*gag*^ to p55^*gag*^ were determined for each mutant and normalized to those for a wt in parallel experiments. Data were obtained from three independent experiments. Error bars indicate standard deviations. *p<0.05; ***p<0.001.

**Fig 6:**
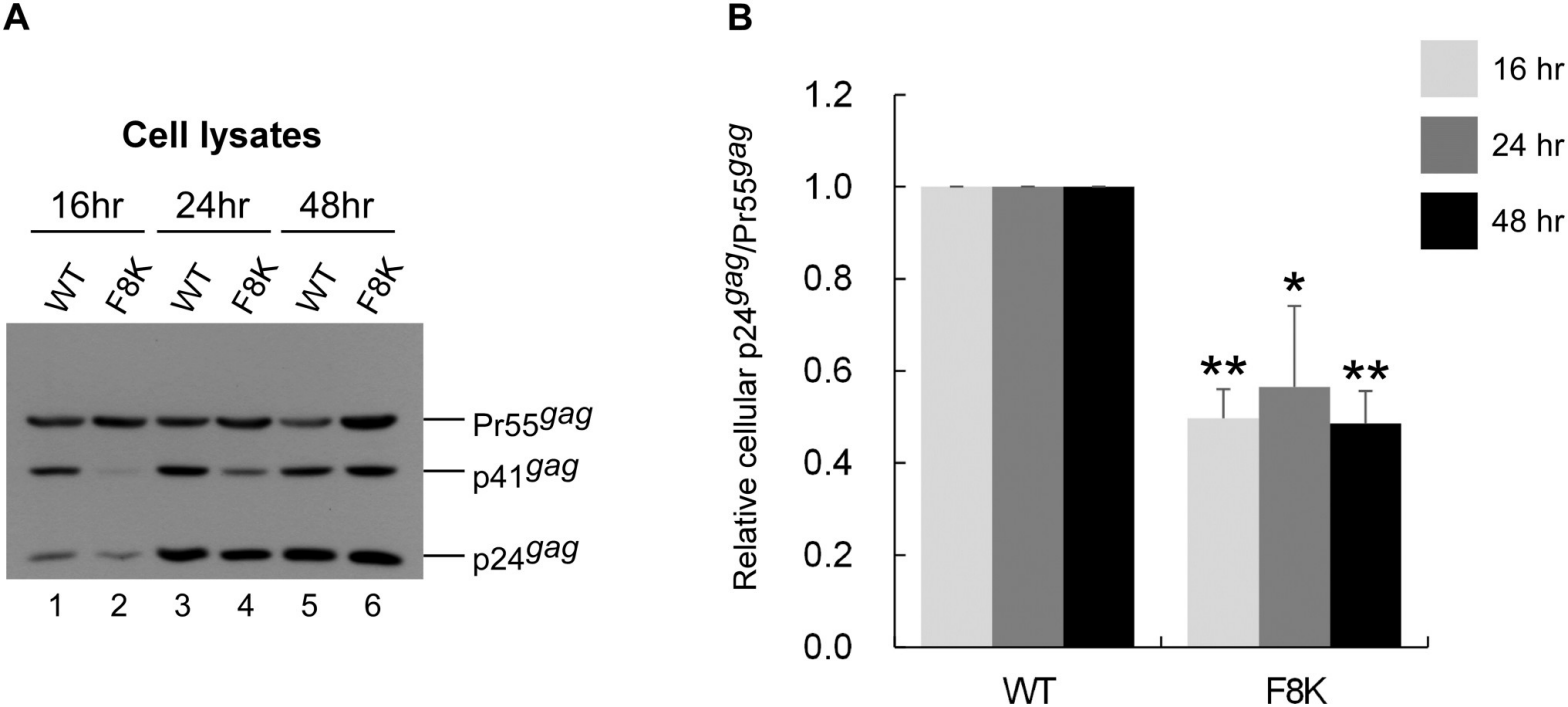
Effects of F8K mutation on Gag cleavage. HEK293T cells were transfected with indicated constructs. At 4 h post-transfection, equal amounts of cells were plated on three dish plates. Cells were collected at 16, 24 and 48 h, and subjected to immunoblot. Panel A is a representative immunoblot from three independent experiments. Cellular Pr55^*gag*^ and p24^*gag*^ levels were quantified by scanning immunoblot band densities. Ratios of p24^*gag*^ to Pr55^*gag*^ were determined and normalized to those of wt in parallel experiments. Bars indicate standard deviations. *p<0.05;**p<0.01 (panel B).

Quantification results for virus particle processing efficiency indicate that both F8V and F8K had significantly lower p24^*gag*^/Pr55^*gag*^ ratios compared to the wt, suggesting a defect in virus maturation (Fig 5). A kinetic analysis of intracellular Gag cleavage indicates F8K had a slower cleavage rate than wt (Fig 6), supporting that mutations at F8 can affect PR-mediated Gag cleavage efficiency. The defects in virus maturation and RT package may partly account for marked reductions in infectivity following mutation substitutions at F8. Since most of the infectivity-impaired mutants we observed exhibited Gag processing efficiency levels comparable to that of the wt, immunoblot assays may be insufficient for detecting subtle but damaging impacts on virus maturation resulting in reduced infectivity. We cannot exclude the possibility that the mutations impaired infectivity during other stages of the virus replication cycle. Combined, our data suggest that mutations at the internal p6* cleavage site can impair virus and PR maturation.

## Discussion

Previous studies have suggested that conserved C-terminal p6* tetrapeptide mutations trigger significant PR maturation impairment [10, 20, 21]. For the present study we focused on analyzing the effects of other conserved p6* residue mutations on virus processing and PR maturation. Since changes in the conserved p6* residues used in this research did not affect p6gag residues, it remains unknown whether the other conserved p6* residues contributed to PR maturation. Although immunoblot data reveal lower virus particle processing efficiency for some p6* mutants (Fig 5), virus-associated RT and PR were readily detected in most mutants. This agrees with a previous report indicating that p6* point mutations did not exert major impacts on the quantitative cleavage of Pol components, despite the possibility that the mutations may have affected Gag cleavage [10]. F8V, F8K and F8I particles contained readily detected p6*-PR (Figs 3 and 4), suggesting that mutations at the internal p6* cleavage site may inhibit the PR maturation process. It is likely that insufficient PR maturation contributes to lower virus processing efficiency, which in turn leads to markedly reduced virus infectivity.

Inhibited PR maturation due to reduced PR activity or an internal p6* cleavage defect may lead to cleavages at cryptic substrate sites [38]. In support of this hypothesis, we found that a 14 kDa PR intermediate was readily detected in wt, F8K and F8I particles following the partial inhibition of PR activity (Fig 4).

Internal p6* cleavages at F8/L9 were determined by observing the autocleaving of synthesized HIV-1 Gag-Pol or p6*-PR expressed and purified from bacterial cells *in vitro* [19, 36, 37, 39–41]. However, to our knowledge the PR intermediate delTFP-p6*-PR derived from a cleavage at F8/L9 is not present in mature HIV-1 virions. A similar product was found within HIV-1 particles following the partial inhibition of PR activity [42]. Results from an analysis of p6* mutation effects on PR activation also failed to detect delTFP-p6*-PR in virus particles [10]. A mutation at F8V has been described as having no major effects on virus infectivity or PR maturation, with mature PR identified as predominant, and with p6*-PR barely detectable [10]. In a separate study, p6*-PR was the predominant PR species, and mature PR was barely detected in virions released from HIV-1-infected human cells [43]. These discrepancies may be due, at least in part, to the employment of different systems. Ludwig et al. concluded that the F8V mutation exerted no major impacts on virus and PR maturation after transiently expressing p6* mutants in an H1299 human lung cancer cell line (2008). During virus assembly and processing, viral polyprotein folding and conformational change may affect target sequence accessibility [44]. Embedded PR release from Gag-Pol entails an initial intramolecular cleavage at the PR N-terminus. The second step, intermolecular cleavage at the PR C-terminus, releases mature PR [39, 41, 45]. Immature PR precursor possesses substantial enzymatic activity [43, 46] and is less sensitive than mature PR to protease inhibition [39]. In our study, p6*-PR precursors were readily detected in F8 mutant particles (Figs 3 and 4), strongly suggesting that mutations at p6* F8 affect PR maturation. It is likely that F8 mutations incur a conformational change that impairs the PR maturation process by destabilizing PR precursor dimer interactions.

## Conclusion

Most of our p6* mutations did not exert major impacts on virus particle processing, but some were found to significantly impair virus infectivity. In particular, we found that mutations at a putative internal p6* cleavage site markedly impaired virus processing and infectivity, likely due in part to defects in PR maturation and RT package. We do not have evidence indicating that a cleavage occurs at F8/L9, but our data strongly suggest that the Phe conserved residue at p6* position 8 is important for PR maturation.

## Supporting information

S1 Raw images This contains all the original blots. (PDF)
