# Supplementary material for: Amino acid substitutions at the HIV-1 transframe region significantly impair virus infectivity
Source: PLoS One. 2022 Jan 27;17(1):e0262477. doi: 10.1371/journal.pone.0262477 (PMC8794111; doi:10.1371/journal.pone.0262477)

Original uncropped blot Fig. 2B

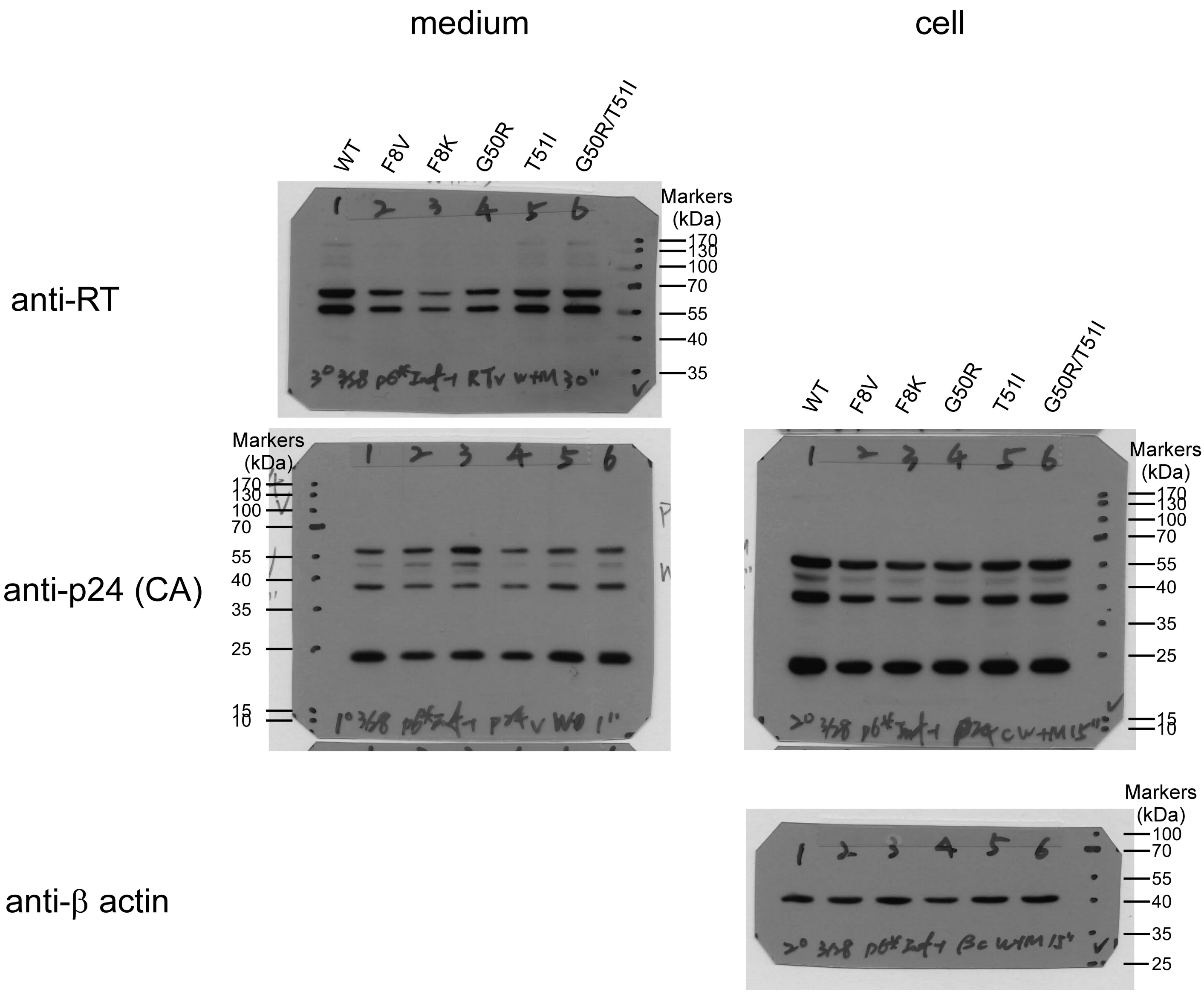

Original uncropped blot Fig. 2C

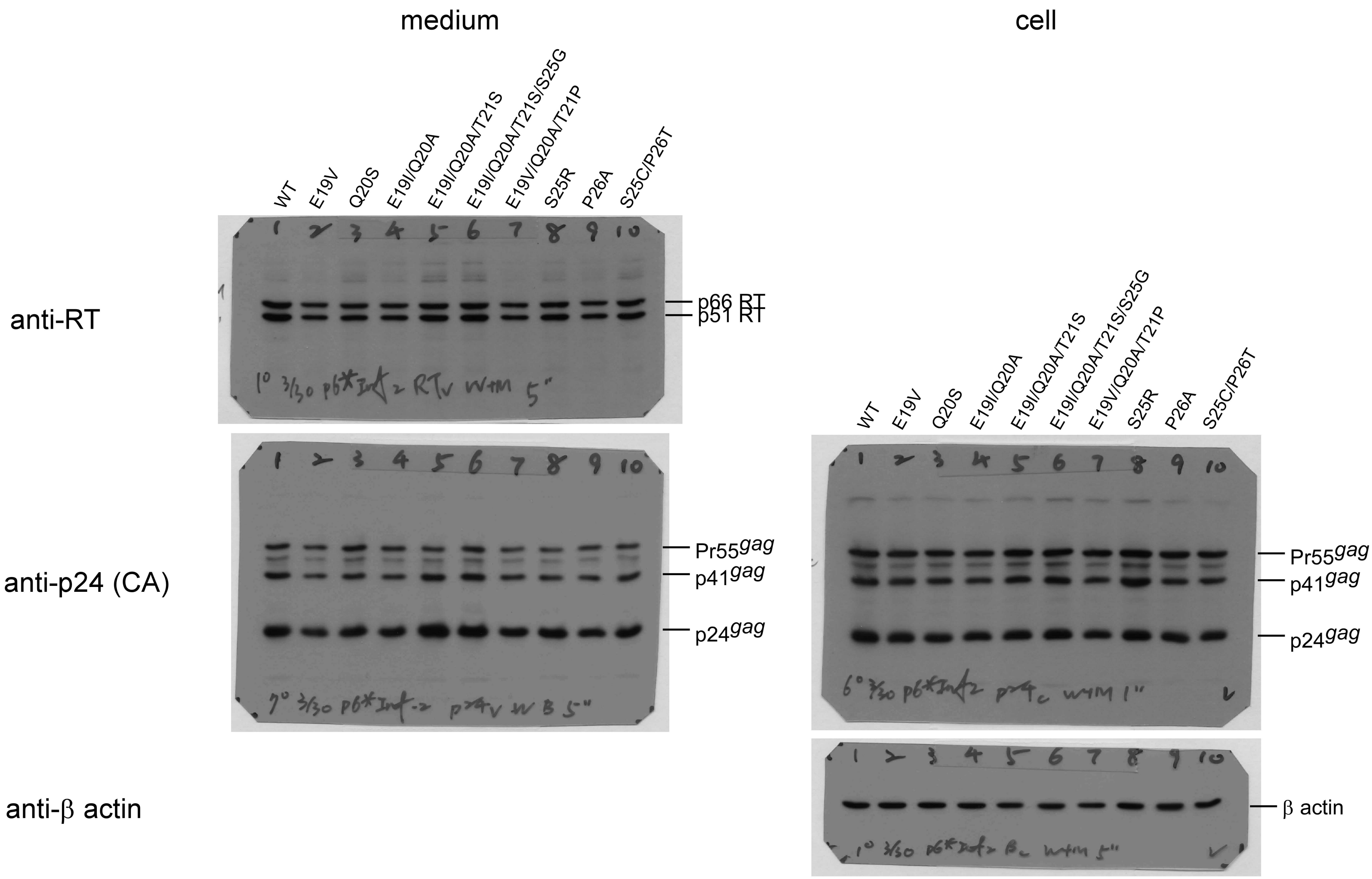

Original uncropped blot Fig. 3A

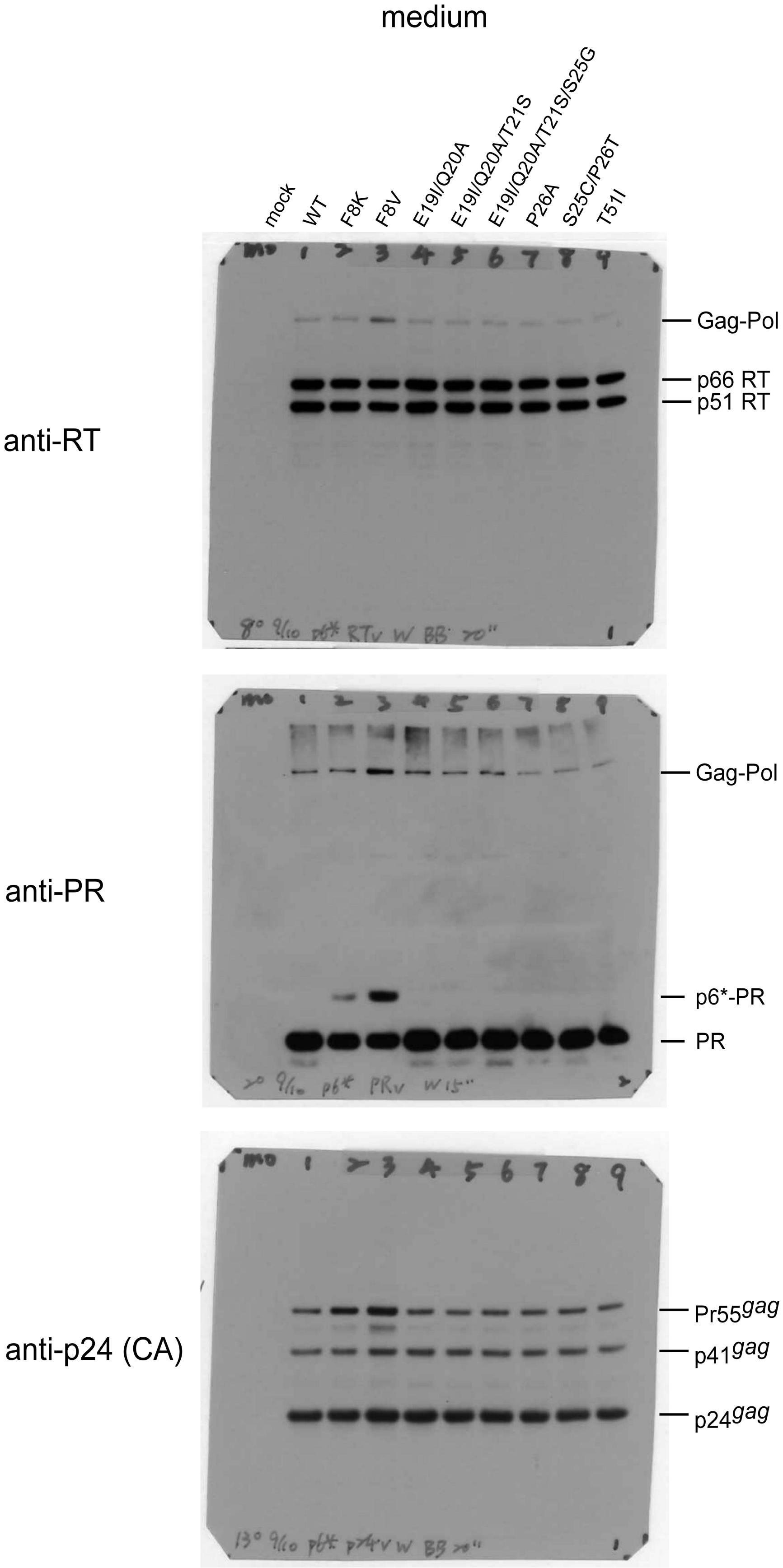

Original uncropped blot Fig. 4

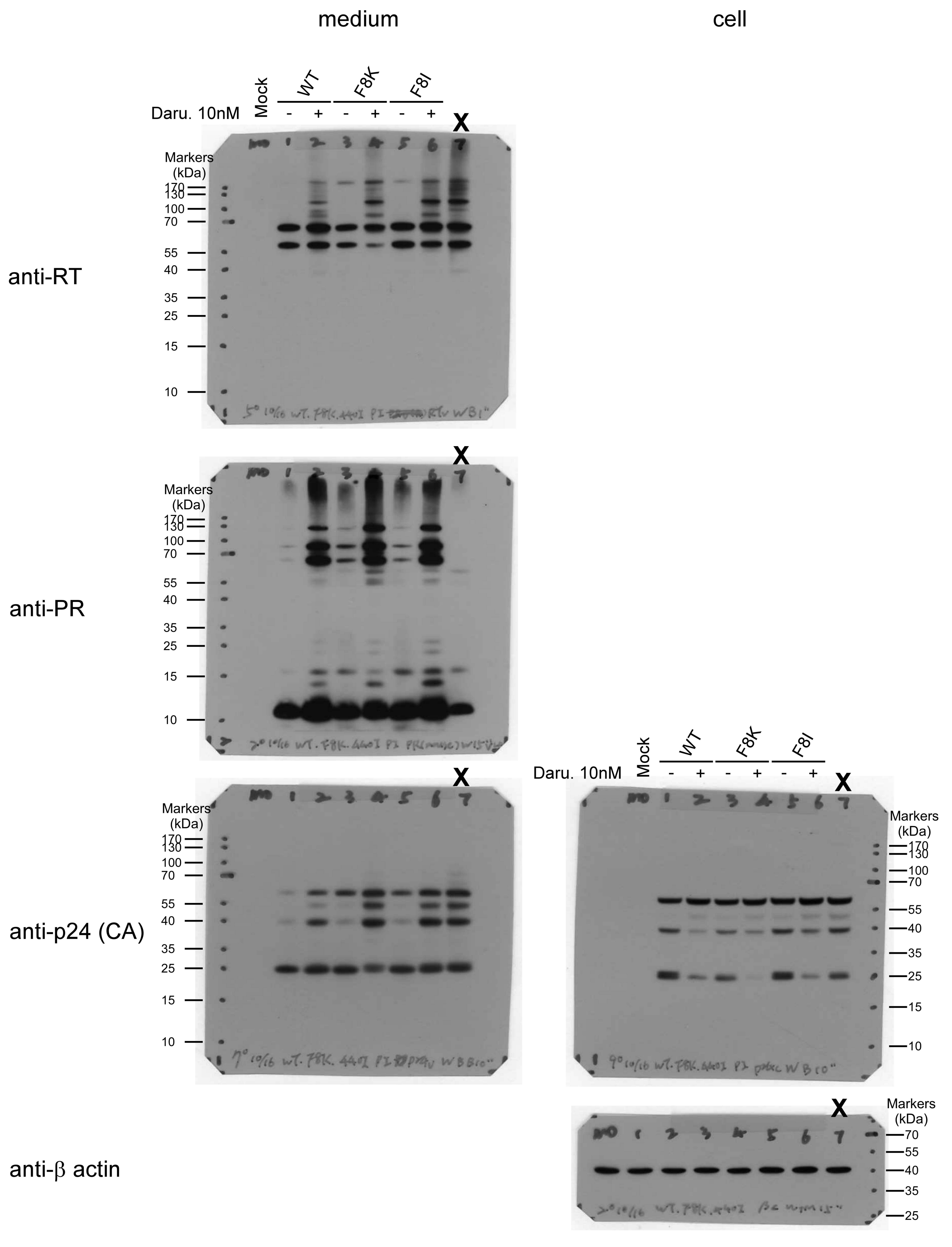

Original uncropped blot Fig. 6A

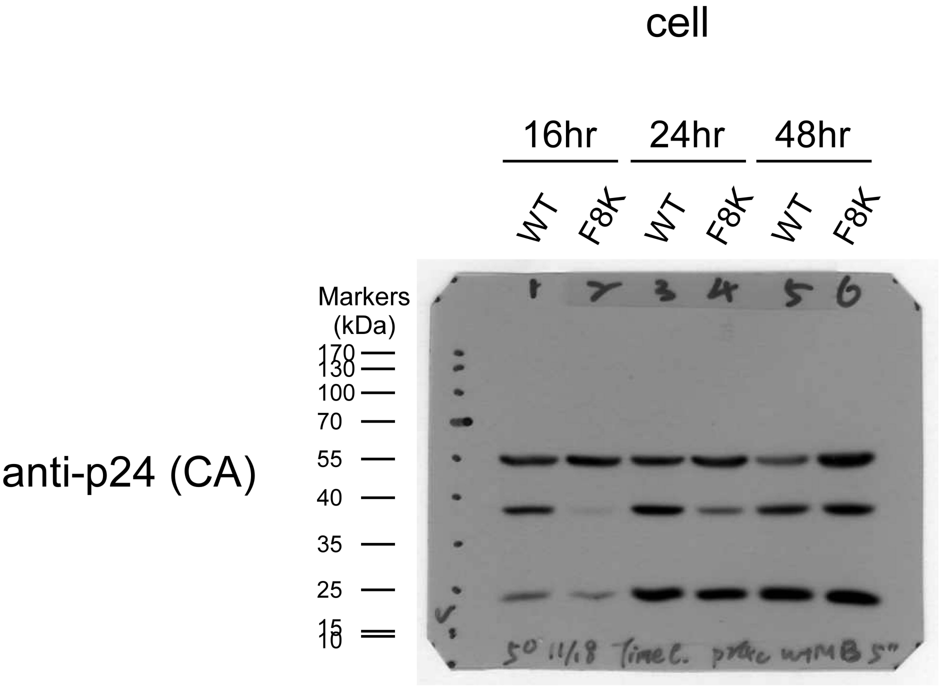

Supplement: S1 Raw images — (PDF) [file pone.0262477.s001.pdf]
